# Medical Association of Georgia

**Published:** 1881-05-20

**Authors:** 


### *MEDICAL ASSOCIATION OF GEORGIA.*

The Medical Association of Georgia convened in Thomasville on the 20th of April last, and, in the absence of the President—the venerable Dr. Joseph A. Eve—was called to order by the Second Vice-President, Dr. B. R. Doster.

The address of welcome was delivered by Dr. T. S. Hopkins, and responded to, in behalf of the Association, by Dr. W. F. Holt, of Macon.

The President elect, Dr. J. C. LeHardy, then took the chair and proceeded to deliver the annual address.

He faithfully reviewed the history of the Association, and deplored the lack, on the part of the profession of the State, of that earnest support to which the organization is entitled and ought to receive at the hands of its members, and at the hands of physicians who are not members of the body.

Among other suggestions he proposed the re-establishment of the committee on prize essays. He had, by his own efforts, raised about one hundred dollars to be devoted to this purpose if a committee should be appointed. This able address will be printed in full in the forthcoming volume of transactions.

The committee to whom the address was referred subsequently reported in favor of the committee on prize essays, which report was adopted by the Association, and the committee was appointed.

Dr. James B. Baird resigned the office of Secretary, which he has filled for the last four years with great faithfulness and ability.

A number of papers were presented during the meeting, and referred to the committee on publication.

A statement was made by Dr. W. H. Hall, member of the Board of Trustees of the State Lunatic Asylum, in reference to the condition of that institution. He urged the necessity for increased room and a more liberal appropriation. The views of Dr. Hall were forcibly indorsed by Dr. Thos. H. Kenan, one of the resident physicians at the asylum.

The question was referred to a committee consisting of Drs. W. H. Hall, Jas. B. Baird and B. R. Doster, with instructions to lay the facts and the necessity for action before the next Legislature.

A bill entitled "An act to establish a Medical Board, to prescribe its purposes, powers and duties, and to regulate and improve the practice of medicine in the State of Georgia, and to provide a penalty for the infringement of the same," was indorsed, and a committee was appointed to urge upon the next L gislature the importance of its passage. We cannot, at this writing, speak advisedly upon this bill, not having seen or heard the details thereof, but as there is already a law of the kind in force in our State, such action would seem to be unnecessary.

Twelve or fifteen new members were added to the roll of membership.

The following officers were elected:

PRESIDENT—Dr. W. F. Holt, Macon.

FIRST VICE-PRESIDENT—Dr. Eugene Foster, Augusta.

SECOND VICE-PRESIDENT—Dr. T. H. McIntosh, Thomasville.

SECRETARY—Dr. A. Sibley Campbell, Augusta.

TREASURER—Dr. K. P. Moore, Forsyth.

CENSOR—Dr. R. J. Nunn, Savannah.

ORATOR—Dr. J. R. Duggan, Macon.

Atlanta was selected as the next place of meeting, which is appointed for the third Wednesday in April, 1882.

A vote of hearty thanks for the kind and courteous attentions bestowed upon the visiting members was enthusiastically passed, and the session was adjourned.

The Association was elegantly entertained by the hospitable people of Thomasville. A sumptuous banquet and a grand ball were given in its honor, so that the social, no less than the scientific features of the reunion were a decided and pleasing success.

